# Unmasking the Molecules Behind our Emotions

**DOI:** 10.1021/acscentsci.5c01268

**Published:** 2025-08-04

**Authors:** Marta Zaraska

## Abstract

Scientists are identifying the chemicals we emit when we emote—and learning how they affect the behavior of people around us.

## Graphical abstract

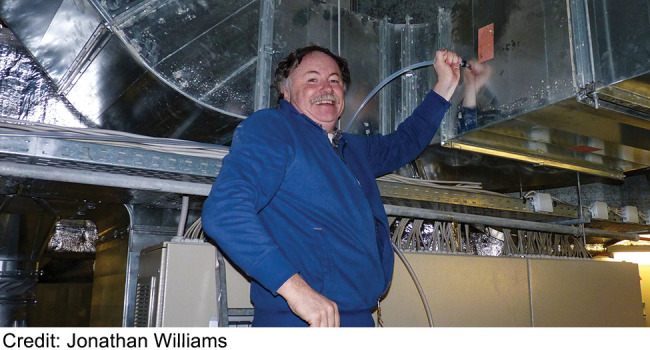

Neurobiologist Shani Agron has been collecting tears for well over a decade. The first step is finding people who cry easily, yet even such “frequent criers” rarely produce more than a few drops per session. At the Weizmann Institute of Science, where Agron works, this involves screening an emotional movie, such as *Titanic*.

The moment the ship goes down, Agron gives the volunteers a pipet to carefully transfer tears from their cheeks into a collection tube. The tubes then go into liquid nitrogen so that the precious odor molecules do not evaporate. Frozen solid, the stock of tears accumulates year after year.

By 2022, Agron had managed to collect enough tears for her experiments. She invited dozens of volunteers into her lab and had them sniff either tears or a saline solution. Right after, Agron ushered the volunteers into functional magnetic resonance imaging (fMRI) scanners and had them play a monetary game designed to measure aggression (they thought they were being robbed by the opposing player). This is when Agron saw her long-awaited results, published in late 2023: sniffing tears tuned down the activity of brain regions associated with hostility and reduced male aggression by over 40%.

“The molecules in tears really affect our emotions,” Agron says.

Research increasingly shows that volatile organic compounds (VOCs) released by one person’s body can be smelled by another and can impact their behaviors. With gas chromatography/mass spectrometry more accessible these days, scientists are working to uncover the exact molecular cocktails that lead us by the nose. Understanding the connections between emotion-produced VOCs and human behavior could allow for practical applications, such as encouraging people to shop faster, keeping employees alert in jobs such as air traffic control, and even facilitating robot-human interactions.

**Figure:**
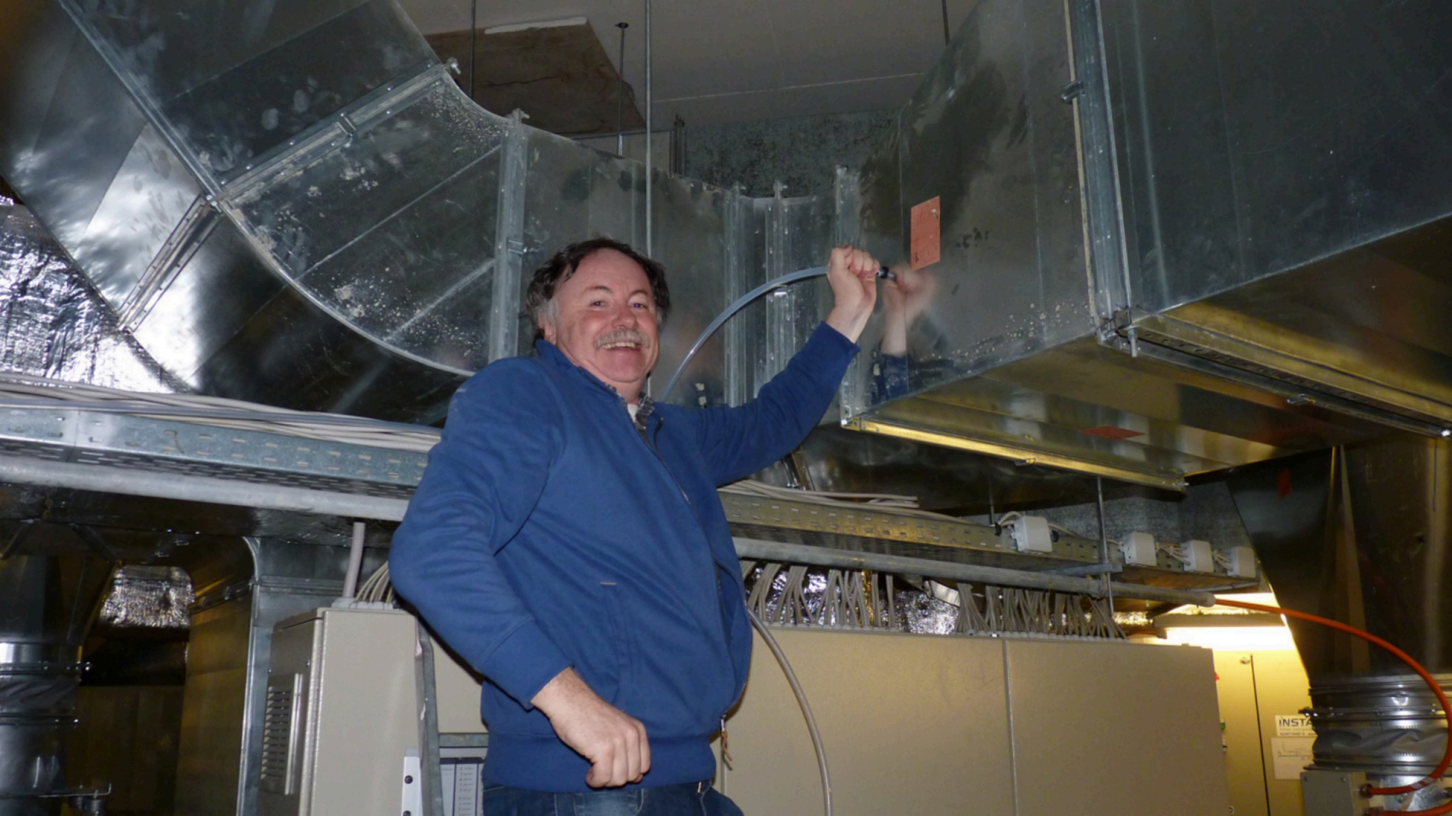
Engineer Thomas Klüpfel connects a cinema’s air vents to an online mass spectrometer to track the volatile organic compounds emitted by the audiences in 2013. Credit: Jonathan Williams.

## The nose knows

Many human body odors waft from under our arms and from our groins. That is where apocrine glands are concentrated, producing all kinds of sweat—including the emotional kind. Initially, those secretions are odorless, but they are transformed by friendly skin bacteria into the VOCs picked up by our noses.

“Evidence suggests that emotional sweat differs from exercise sweat, both in terms of its effect on behavior and chemical composition,” says Helene Loos, a chemist and smell researcher at Friedrich Alexander University Erlangen-Nuremberg.

But it is not only the apocrine glands that create body odor—be it the emotional kind or the kind you get from, say, exercising. Hundreds of molecules are emitted from our skin, from esters and ethers to alcohols, that together create a unique human smell that in one study perfumers described as heavy, milky, metallic, and grasslike (with men apparently smelling more metallic than women).

Glycoproteins and sugars flow out from eccrine glands concentrated in the forehead, hands, and feet but generally present across the whole body. Lipids emerge from our sebaceous glands, found in the highest densities on the upper torso and scalp. And that is far from all the compounds contributing to body odor, many of which have not even been identified.

“These substances are really, really low in concentration, and it’s pretty hard to properly identify the compounds,” says Andrea Büttner, a chemist at Friedrich Alexander University Erlangen-Nuremberg.

While scientists use gas chromatography/mass spectrometry to detect the molecules of body odor, human noses are quite adept at this skill, too. Contrary to popular belief, “We’re not necessarily inferior to other species when it comes to our sense of smell,” says Jasper de Groot, a behavioral psychologist at Radboud University. Some studies suggest we can discriminate over 1 trillion olfactory stimuli. Others show that we can follow a trail of scent across a lawn, like a tracking dog. We can detect the smell of a single fly in a glass of wine. We can even tell whether a fellow human is sick. Most mothers are able to recognize their child’s smell as soon as 10 min after birth. Using body odor, many people can tell family members from strangers, match identical twins, and pick potential friends. That is no magic: human body odors depend in part on our genetics, and no two people smell the same.

Our noses also have a more specific superpower: picking up the emotions of others, which, in turn, changes our own emotions and behaviors. In one early study, scientists approached dozens of people scheduled for their first skydive. During the 13,000 ft descent, to collect sweat, the volunteers wore absorbent pads taped under their arms. The contents of the pads were later converted into a fine mist with a nebulizer and presented for sniffing to another group, alongside control sweat from people who exercised on a treadmill.

After taking a whiff, the volunteers were asked to look at photos of male faces. Using image-morphing software, the researchers generated a series of facial expressions along a range from neutral to angry. Those who had smelled the anxious skydiving sweat, on average, interpreted the faces as more angry than the control group did. What’s more, on an fMRI scan, their amygdalae—the fear centers of the brain—lit up.

To show that odors of emotions can infect others in less extreme scenarios, de Groot and his colleagues studied sweat from underarm pads worn by people who watched movie scenes chosen to induce fear (like from *Misery*, a horror film), happiness (*The Jungle Book*), and neutral states (a video of a train traveling through the Alps). When the odors were sniffed by people who had electromyography electrodes attached to their faces, the sensors picked up subtle differences in facial muscle activity: in those who smelled the happy sweat, the eyelid muscles twitched more, indicating a smile, while in people who inhaled fear odors, the medial frontalis muscle was activated, raising the eyebrows. From an evolutionary perspective, it makes sense, de Groot says: “You open your eyes to better detect threats.”

**Figure:**
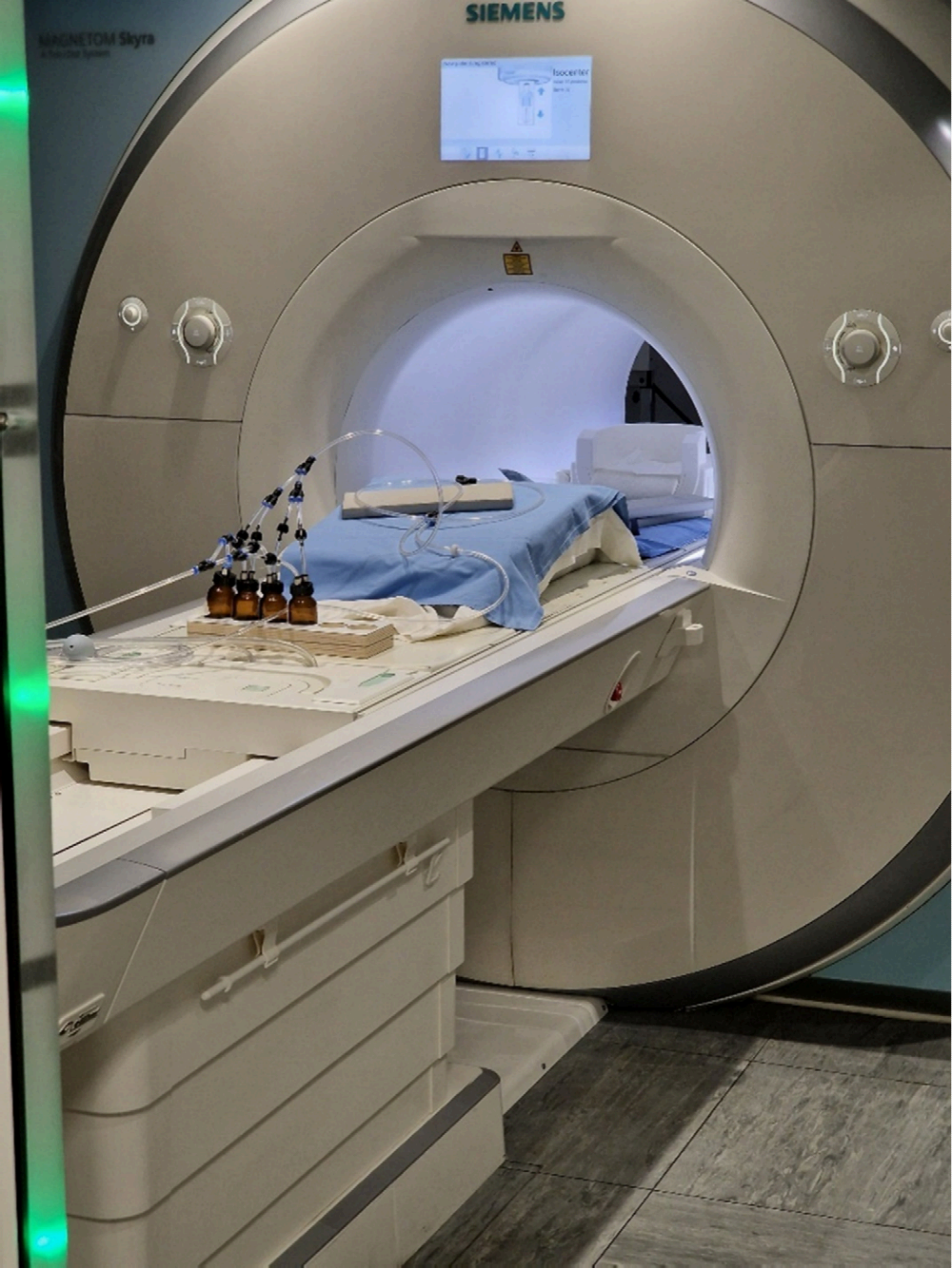
Functional magnetic resonance imaging machine setup for Jasper de Groot’s experiments on how emotional sweat affects brain activation. Credit: Jasper de Groot.

Molecules emitted by our bodies can have an impact even if we do not consciously realize they are present. Jan Havlíček, an evolutionary psychologist at Charles University, says that in one “really neat” experiment, dentistry students had to perform surgery on a practice mannequin. In some scenarios, the mannequin wore a T-shirt infused with anxious human sweat, while in others, the shirt was clean. To mask any potential stinkiness, all shirts were soaked in eugenol, a typical scent of dentistry offices. While the students claimed they could not smell anything different, they did perform differently: the quality of their work went down by over 18% when the mannequin was wafting VOCs of anxiety. In other words, you do not want your dentist to smell your fear.

## Uncovering the chemicals we emit

With so many studies showing that other people’s body odors can affect our behavior, scientists have started looking for the molecules responsible for these effects.

“Research turned from psychology and neuroscience to chemical analysis,” de Groot says.

One of the first such analyses was done in a German cinema that was screening three different movies: *The Hunger Games: Catching Fire*, a dystopian action movie; *Walking with Dinosaurs*, a family film shown in 3D; and *Buddy*, a comedy. Unbeknownst to the people in the audience, scientists connected the cinema’s air vents to a proton-transfer-reaction mass spectrometer, which analyzed the levels of VOCs floating out of the theaters. Such measurements were conducted over 108 screenings while the audiences watched the movies as usual.

When the researchers reviewed the results, they found that isoprene, a small, volatile molecule with a slightly musty aroma, spiked during certain scenes of *The Hunger Games*—for instance, when Katniss Everdeen’s dress catches fire during the tribute parade. Such spikes, however, did not happen during *Walking with Dinosaurs* or *Buddy*. Since isoprene is linked to the production of cortisol, a stress hormone, the scientists could basically plot the anxiety-inducing scenes of *The Hunger Games* simply by looking at the isoprene levels in the theater’s air vents.
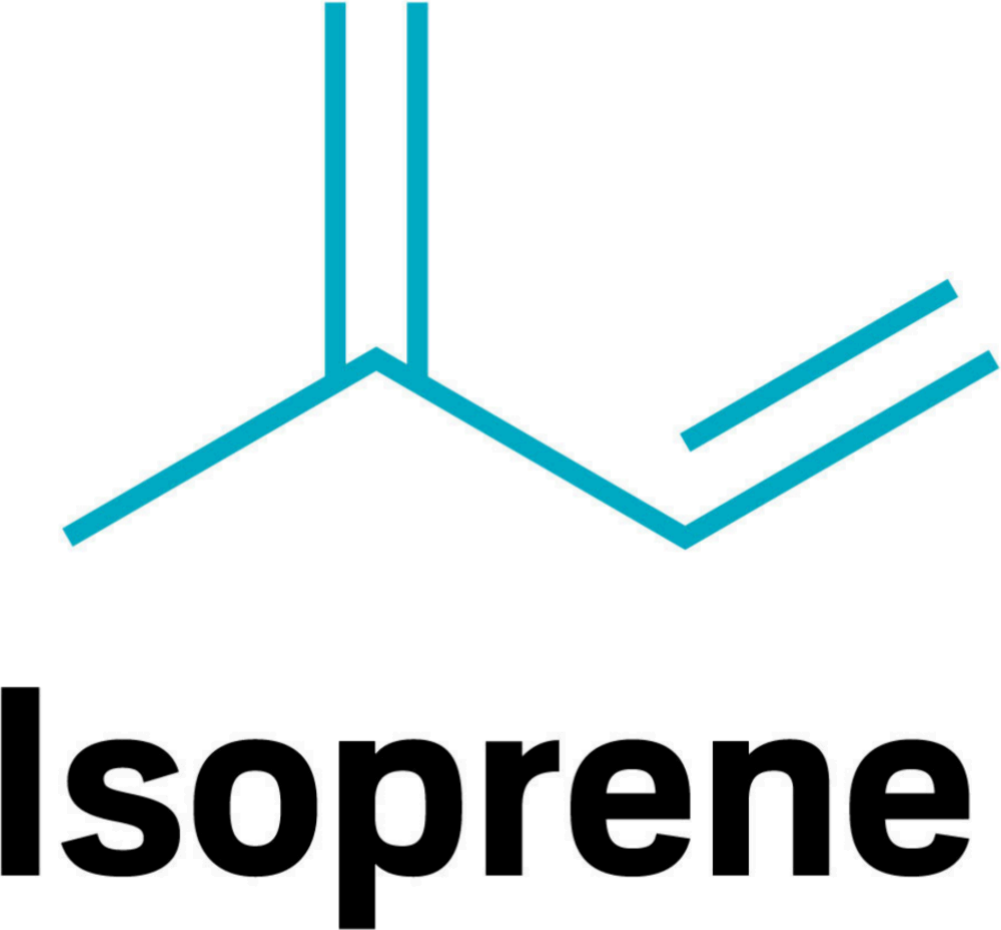


While such real-world studies can be insightful, they are “really expensive to do,” de Groot says. In those settings, it is also difficult to control all the variables, such as dust in the air or the cosmetics people wear. So de Groot and his colleagues look for molecules of contagious emotions in the lab. One of the first ones they focused on was hexanal, a grassy-smelling compound found in body odor. “It’s also present in a lot of scents that are typically used to calm people down, like lavender,” de Groot says. This gave him the idea that hexanal might have something to do with soothing emotions.
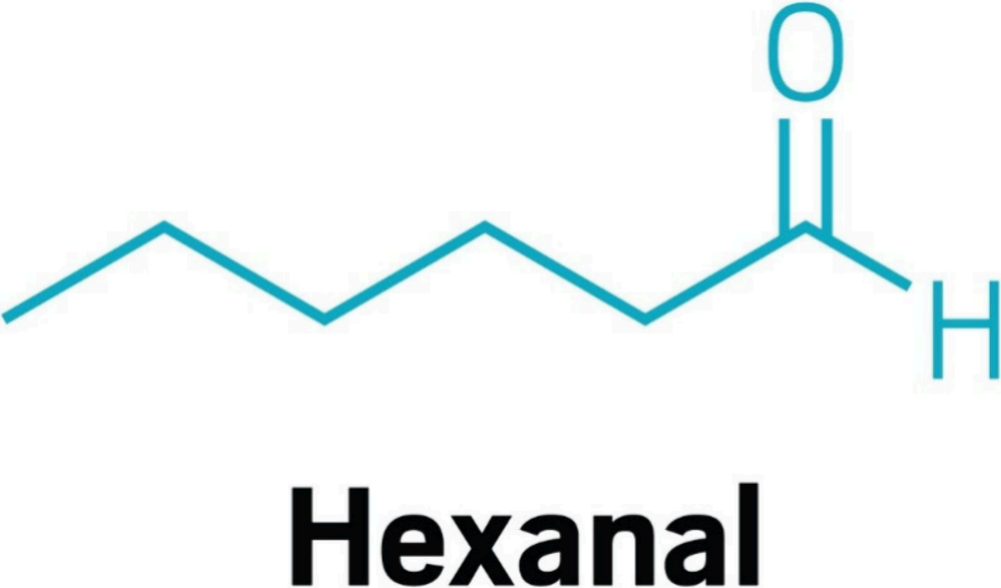


He found that when volunteers sniffed hexanal, even when it was masked by eugenol, they trusted each other more in a money game. De Groot warned, however, that the results were preliminary and he does not “want to bet full money on it.” We still do not know, for instance, if people who have more hexanal in their body odor are perceived as more trustworthy, says Monique Smeets, a social and cognitive psychologist at Utrecht University and de Groot’s coauthor. “It is not an easy test but certainly interesting,” she says.

De Groot is more confident about his more recent research: together with Smeets and other colleagues, he collected sweat from people watching various emotional videos, extracted VOCs from the underarm pads, and analyzed the VOCs using gas chromatography/mass spectrometry. They discovered that fear sweat was flush with aldehydes and ketones but low in esters and cyclic molecules—the exact opposite of neutral sweat from people who watched a video clip of a moving train.

**Figure:**
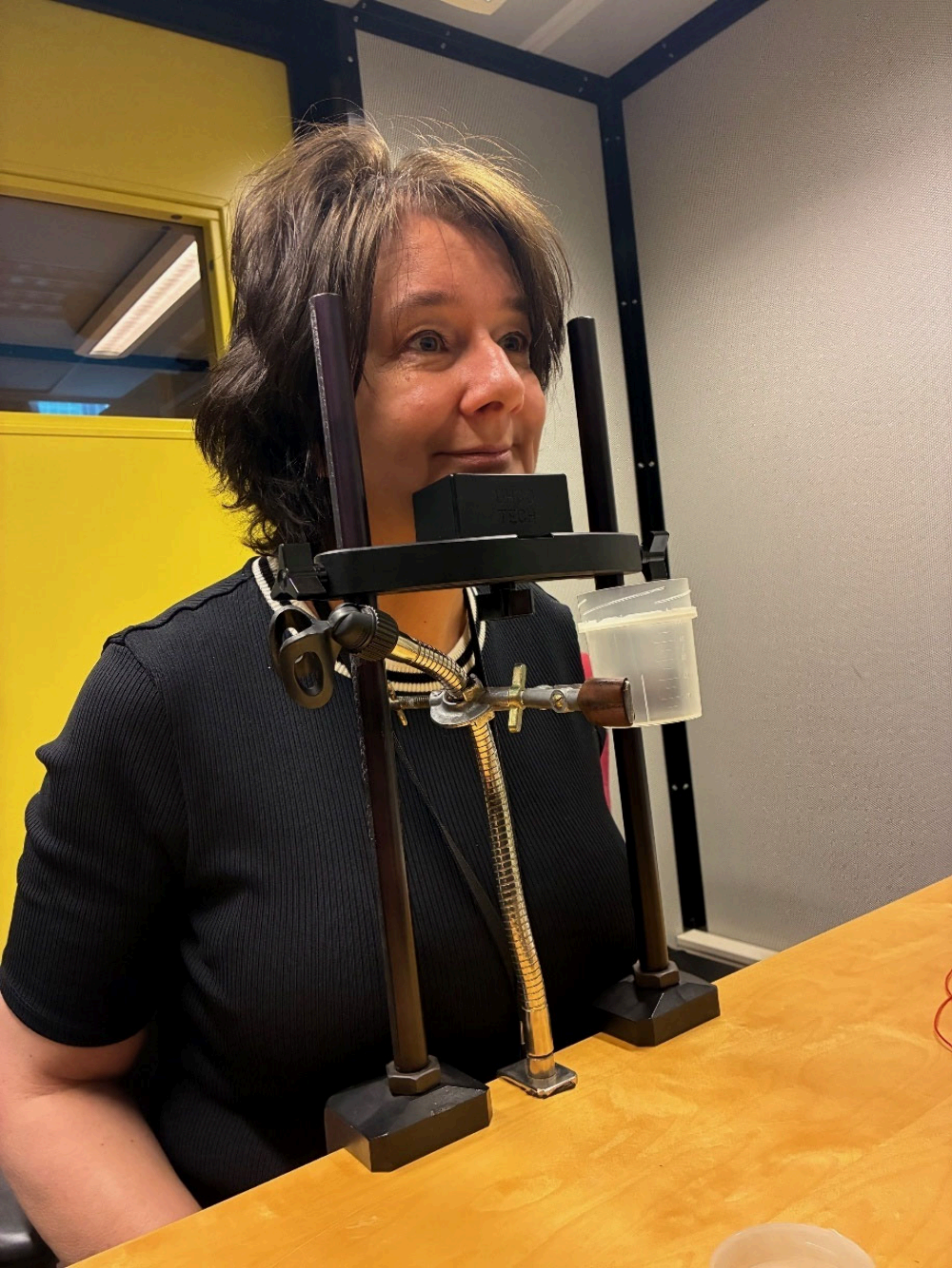
Social and cognitive psychologist Monique Smeets testing the setup for smelling a pad containing body odor. Credit: Hans Marien.

Next, in a follow-up study, de Groot and his colleagues zeroed in on specific molecules. “We saw that it’s mostly the carboxylic acids that are in higher abundance in fear—for instance, hexadecanoic acid and tetradecanoic acid,” he says. Also in that study, the researchers showed that such fear-related molecules are universal and are present even in people who, because of a genetic mutation, have almost odorless sweat, a phenotype most common in those of East Asian descent.

Yet, most likely, no single molecule is responsible for fear or happiness contagion. “It’s more of a cocktail of different molecular ingredients,” de Groot says.

## The future of emotional emissions

Once we figure out which exact cocktail of compounds is driving the spread of each emotion, we may be able to use this knowledge in applications such as robotics or long-distance communication. Electronic noses can already pick up molecules indicating certain diseases, such as diabetes, and recent studies show that it is also possible to measure stress and fatigue based on VOCs emitted by our bodies. One can imagine that future machines might be able to interpret our emotions based on body odor, Büttner says.

Researchers are already investigating applications involving human interaction with robots. A 2025 study showed that when Nao, a French humanoid robot, wore a necklace wafting estratetraenol, a compound produced by female bodies and thought to encourage cooperation, people trusted the robot more. (But in another study, releasing hexanal from robots didn’t work.)

There are many ways to imagine how emotion-signaling chemicals could be used for humanity’s benefit (just picture a Zoom call where you can be infected by your friend’s smell of happiness), but Havlíček worries that this technology may have a dark side. “It could be used, for instance, as a biological weapon, creating panic,” he says. “It’s quite challenging to think ahead with these kinds of things,” he admits.

Some potential applications fall into a moral gray area. For example, molecules of emotions could be used in commerce. One recent study showed that consumers make their shopping decisions faster when they can smell human body odor.

On the other hand, we might be able to use emotion-related molecules to make life safer. In one study, the odor of fear reduced inattentional blindness: after taking a sniff of a person who had experienced fear, people could more easily spot a shark suddenly appearing in a virtual aquarium. The study’s authors suggest this might have potential applications for people carrying out “continued-vigilance tasks,” such as air traffic controllers.

In certain jobs, it appears, it may be a good idea to follow your nose.

*Marta Zaraska is a freelance contributor to* Chemical & Engineering News, *the independent news publication of the American Chemical Society.*

